# Job vacancy at the European Centre for Disease Prevention and Control (ECDC)

**DOI:** 10.2807/1560-7917.ES.2024.29.1.2401047

**Published:** 2024-01-04

**Authors:** Eurosurveillance editorial team

**Affiliations:** 1 European Centre for Disease Prevention and Control (ECDC), Stockholm, Sweden

The European Centre for Disease Prevention and Control (ECDC) invites applications for the following position:

 

**Principal Expert Microbial Safety of Substances of Human Origin**
The application deadline expires on 31 Jan 2024.

 

For more information, please visit the ECDC website: https://erecruitment.ecdc.europa.eu/?page=advertisement

